# Cost-Utility analysis of Patient-Led Follow-Up after Rectal Cancer Compared to Standard Follow-Up: A three-year follow-up of the FURCA Randomised Controlled Trial

**DOI:** 10.1007/s11764-026-01976-9

**Published:** 2026-01-29

**Authors:** Bettina Wulff Risør, Nasrin Tayyari, Liza Sopina, Therese Juul, Søren Laurberg, Henriette Vind Thaysen, Ida Hovdenak

**Affiliations:** 1 DEFACTUM, Central Denmark Region, Olof Palmes Allé 15, Aarhus N, 8200 Denmark; 2 Danish Center for Health Economics, University of Southern Denmark, Campusvej 55, Odense C, 5230 Denmark; 3 Department of Surgery, Aarhus University Hospital, Palle Juul-Jensens Boulevard 99, Aarhus N, 8200 Denmark; 4 Department of Clinical Medicine, Aarhus University, Palle Juul-Jensens Boulevard 82, Aarhus N, 8200 Denmark

**Keywords:** Rectal cancer, Follow-up care, Patient-led, Cost-utility analyses, Health economics, Randomised controlled trial, Survivorship

## Abstract

**Purpose:**

Rectal cancer survivors are at risk of late adverse effects that impair quality of life. This study evaluates the cost-utility of a patient-led follow-up programme introduced in the Danish FURCA randomised controlled trial (RCT), aimed at improving patient outcomes and optimising healthcare resource use compared to standard hospital-based follow-up.

**Methods:**

The cost-utility analysis was performed from a societal perspective over a 3-year horizon, incorporating healthcare costs, prescription drug use, productivity losses, all derived from Danish register data, and quality-adjusted life years (QALYs) derived from RCT-collected EQ-5D-5L data. Incremental cost-effectiveness was assessed using regression models and non-parametric bootstrapping, with subgroup and sensitivity analyses exploring heterogeneity in outcomes.

**Results:**

A total of 336 patients were randomised to intervention and control groups. Over 3 years, mean healthcare costs were €40,208 for the intervention group and €41,190 for the control group (difference −€980; 95% CI −€7120 to €5159). Mean QALYs were 2.24 and 2.20, respectively (difference 0.028; 95% CI −0.106 to 0.163). The incremental cost-effectiveness ratio was −€35,048 per QALY gained, indicating dominance. Scatterplots of bootstrapped incremental cost-effectiveness ratios (ICERs) revealed iterations in all four quadrants, reflecting substantial uncertainty in both costs and effects. The probability of cost-effectiveness at a €30,000 threshold was below 70%.

**Conclusions:**

Patient-led follow-up resulted in comparable QALYs and costs relative to standard hospital-based follow-up. The probability of cost-effectiveness at a conventional willingness-to-pay threshold reached up to 70%.

**Implications for cancer survivors:**

Patient-led models may enable tailored delivery of specialist care to patients with greatest need, alongside balanced resource utilisation.

**Supplementary information:**

The online version contains supplementary material available at https://doi.org/10.1007/s11764-026-01976-9.

## Introduction

Rectal cancer (RC) represents a substantial global health burden, with incidence increasing in many countries [1]. Although the incidence in Denmark is decreasing, more than 1100 persons were diagnosed with RC in 2023 [2, 3]. Due to considerable improvements in early detection and treatment, recent decades have shown a remarkable decrease in mortality from RC [4, 5]. This development has led to an increasing number of RC survivors, experiencing late adverse effects such as bowel, urological, and sexual dysfunctions, psychological distress, and fear of recurrence, impacting long-term quality of life (QoL).

Traditional RC follow-up programmes have mainly focused on the detection of recurrent disease, although the value of these methods has been questioned due to a lack of consistent evidence [6]. Meanwhile, increasing attention has been directed towards the high burden of late adverse effects following RC treatment, and recent studies suggest that patients may benefit considerably from earlier detection and management of these complications [7–10]. This underscores the need for a broader approach to follow-up and survivorship care after RC treatment, with greater emphasis on detecting and managing late adverse effects. Against this background, alternative models of follow-up have been called for [11–13].

Follow-up after cancer treatment is often resource-intensive due to its long-term nature and the need for coordinated input from multiple health professionals and care settings. Therefore, it is necessary to evaluate the cost-effectiveness of the range of follow-up strategies to ensure that they provide an efficient use of scarce resources [14].

Only limited research is available to evaluate the costs of colorectal cancer (CRC) follow-up [15–18]. A systematic review from 2022 was aimed at examining the individual cost components of treating CRC [18] and follow-up costs were explicitly described in only one of the included studies [19]. In an economic evaluation of data from a randomised trial, Vos et al. found that GP-led care was likely to be cost-effective for disease-specific QoL but not for general QoL [16]. In an observational study of remote follow-up after CRC compared to standard hospital follow-up, Qaderi et al. found that remote follow-up for the total cohort led to €22,408 cost savings over 1 year [17]. Patient-led follow-up was introduced for low-risk endometrial cancer patients and proved to be financial- and timesaving compared to standard hospital follow-up [20] but to date, no economic evaluations have been published examining the efficiency of patient-led follow-up after RC, using data directly arising from a randomised controlled trial with long-term follow-up.

In 2016, a Danish multicentre trial was initiated, introducing a new patient-led follow-up programme for RC survivors (Follow-Up after Rectal Cancer—FURCA) [21]. In a randomised, controlled design, a traditional follow-up model was compared to an experimental, patient-led follow-up programme.

It was assumed that the intervention would empower patients to play an active role in their care pathway, leading to timely management of late adverse effects and signs of recurrent disease. Thus, the patient-led follow-up programme was tested under the hypothesis that the new programme would lead to reduced symptom burden and improved QoL after 3 years of follow-up.

The two different follow-up programmes in this trial are likely to result in systematic variations in patterns of health care utilisation. Some of the variation would be directly explained by a priori defined service differences in the two allocation groups. Other variations may be due to indirect effects of the intervention, such as altered use of general practitioners, other medical specialists, or differences in medical consumption.

The standard hospital-based follow-up period for RC patients in Denmark is 3 years, and it is relevant to evaluate costs related to the effects when elements in follow-up programmes are altered.

It was hypothesised that the new patient-led programme would optimise the use of healthcare resources, mostly due to the relocation of time consumption from surgeons (at clinical visits) to specialist nurses handling patient education and self-referrals [21].

This study is aimed at evaluating the cost-utility of a new patient-led follow-up programme for rectal cancer patients compared to traditional follow-up.

## Methods

### Study design and setting

This is a cost-utility analysis conducted alongside the Follow-Up after Rectal Cancer (FURCA) trial, a multicentre, parallel-group randomised controlled trial. The population-based trial was designed to compare a patient-led follow-up programme with standard surgeon-led follow-up among patients who had undergone curative-intent resection for rectal cancer. In the new programme, up to five routine clinical doctor visits were replaced by patient-led follow-up, based on standardised patient education and patients’ access to direct self-referral to a specialist nurse. This self-referral system was supported by standardised response algorithms to be utilised by the specialist nurse. Recruitment took place between March 2016 and August 2018 at four surgical departments located in the Central and Northern Denmark Regions. The economic evaluation was performed from a societal perspective, in line with national and international guidelines. The study follows the Consolidated Health Economic Evaluation Reporting Standards (CHEERS-2022) [22], and the completed CHEERS checklist is available in Supplementary File 1.

### Participants and allocation

Eligible participants were adults recently discharged after rectal cancer surgery with curative intent. Patients were excluded if they were unable to participate in follow-up due to severe comorbidity, cognitive impairment, or insufficient Danish language skills. A total of 336 participants were enrolled and randomly allocated in a 1:1 ratio to either the intervention or control group using a computer-generated randomisation sequence. Allocation was stratified by hospital site, sex, and treatment type. Blinding of participants and clinical staff was not feasible due to the nature of the intervention. Details of the study design of the FURCA trial are published elsewhere [21].

### Intervention

In the intervention group, routine outpatient visits were replaced by a patient-led follow-up model. This included a structured education session before discharge, aimed at enhancing patients’ ability to monitor symptoms and act on health concerns. Participants were granted direct access to a specialist nurse through self-referral, and the nurses responded using algorithm-based protocols developed for the trial [21]. The model was intended to promote timely detection and management of both recurrent disease and late adverse effects, while enhancing patient involvement and self-management.

The comparator was a standardised surgeon-led follow-up programme, consisting of up to five scheduled outpatient visits at six-month intervals. Both groups received identical access to surveillance imaging and colonoscopy, in accordance with Danish national guidelines [23, 24].

### Time horizon and perspective

The primary time horizon was 3 years post-surgery, corresponding to the formal follow-up period in both trial arms. The analysis was conducted from a societal perspective, encompassing direct healthcare costs, medicine costs, and productivity losses.

### Measurement of outcomes

The primary outcome for the economic evaluation was quality-adjusted life years (QALYs). Health-related quality of life was assessed at baseline, 1 year, and 3 years after surgery using the EQ-5D-5L instrument. EQ-5D-5L index values were derived using the Danish value set [25]. QALYs were calculated by estimating the area under the utility curve using linear interpolation between observations, whereby health utility was assumed to change linearly between consecutive EQ-5D-5L assessments and accumulated over the follow-up period. Life years were therefore captured implicitly through observed follow-up time, without extrapolation beyond the trial period. To adjust for time preferences, the QALY estimates were discounted at a rate of 3.5%.

### Resource use and costing

Resource use data were obtained from Danish national health and social registers. Primary care contacts were identified through the National Health Insurance Service Register and valued using tariffs [26]. Data on hospital contacts and admissions were retrieved from the National Patient Register, with costs estimated using national average Diagnosis-Related Group (DRG) tariffs [27]. Dispensed prescription drugs’ costs were obtained from the Danish National Prescription Registry and valued using actual retail prices [28].

Productivity losses were calculated using the DREAM database, which records weekly information on absence from work [29]. Loss of productivity was quantified for working-age individuals who did not reach the statutory retirement age during the study period and valued using the human capital approach [30].

All cost estimates were adjusted to 2024 prices using the Danish Consumer Price Index (CPI). Moreover, all costs were discounted at an annual rate of 3.5%, in line with recommendations from the Danish Ministry of Finance and then converted to euros using the exchange rate of 1 EUR = 7.46 DKK.

### Statistical and economic analysis

Descriptive analyses were used to compare baseline characteristics between the two groups. For continuous variables, group differences were tested using independent samples *t*-tests, and for categorical variables, *χ*^2^ tests were applied. These tests were conducted to assess the comparability of the groups and to inform decisions regarding covariate adjustment in the outcome analyses.

Resource use and costs during follow-up were summarised as mean values per group. Estimates include participants with no utilisation of specific services during the follow-up period, such that zero utilisation and zero costs were retained in the calculation of group-level means.

For the calculation of QALYs, missing EQ-5D-5L index values at baseline were imputed using the mean baseline index value across both groups. For subsequent time points, missing index values were imputed using multiple imputation based on relevant population characteristics; the associations between these characteristics and utility were evaluated before inclusion in the imputation model. Multiple imputation was performed according to Rubin’s rules to appropriately reflect imputation uncertainty [31].

To estimate differences in total costs and QALYs between groups, we applied linear regression models with robust standard errors. The QALY analysis was adjusted for baseline utility to account for potential differences at inclusion [32]. Further covariates were only included if group imbalance was identified in baseline characteristics. Due to the skewed distribution of both cost and QALY data, non-parametric bootstrapping with 10,000 replications was used to derive 95% confidence intervals for mean estimates and between-group differences. This approach provided robust estimates under distributional uncertainty [33–35].

Incremental cost-effectiveness ratios (ICERs) were calculated by dividing the difference in mean costs by the difference in mean QALYs. Uncertainty surrounding cost and effect estimates was assessed using non-parametric bootstrapping with 10,000 replications. The distribution of bootstrapped ICERs was visualised on cost-effectiveness planes, and cost-effectiveness acceptability curves (CEACs) were used to estimate the probability of cost-effectiveness across a range of willingness-to-pay thresholds.

### Subgroup and sensitivity analyses

Various subgroup analyses were conducted to explore heterogeneity in cost-effectiveness by surgical procedure (total mesorectal excision (TME), partial mesorectal excision (PME), and abdomino-perineal resection (APR)) and by receipt of neoadjuvant treatment (yes vs no). For each subgroup, separate ICERs, CEACs, and uncertainty intervals were estimated using the same analytic approach.

All analyses were conducted using Stata version 18.

## Results

### Study population

A total of 336 patients with rectal cancer were enrolled in the trial and randomised equally to either the intervention group (*n* = 168) or the control group (*n* = 168). The flowchart of inclusion and follow-up has been published previously [36]. The mean age at baseline was 65.2 years (SD 8.0) in the intervention group and 65.6 years (SD 9.9) in the control group. The distribution of sex, marital status, and clinical characteristics was comparable between the groups (Table 1). Approximately one-third of participants were female, and the majority were married or cohabiting.

**Table 1:** Baseline characteristics

	Intervention	Control
	(*n* = 168)	(*n* = 168)
Age (years), mean (SD)	65.17 (7.96)	65.57 (9.89)
Gender, *n* (%)		
Female	54 (32)	56 (33)
Male	114 (68)	112 (67)
Marital status, *n* (%)		
Unmarried	14 (8)	14 (8)
Married (or separated)	118 (70)	117 (70)
Divorced	24 (14)	24 (14)
Widowed	12 (7)	13 (8)
Education, *n* (%)		
Short	51 (30)	49 (29)
Medium	108 (64)	113 (67)
Long	9 (5)	6 (4)
Area of residence, *n* (%)		
Densely populated area	37 (22)	44 (26)
Medium populated area	37 (22)	47 (28)
Sparsely populated area	94 (56)	77 (46)
Comorbidity*, *n* (%)		
Mild	149 (89)	157 (93)
Moderate	13 (8)	6 (4)
High	6 (4)	5 (3)

*SD* standard deviation

*Comorbidity calculated based on Charlson Comorbidity Index

### Resource use

Over the 3-year follow-up period, resource utilisation was similar with no significant differences between the intervention and control groups across all measured domains, including primary care, specialist services, hospital-based outpatient and inpatient care, prescription medicines, and productivity loss (Table 2). These findings indicate that the patient-led follow-up programme was not associated with increased resource use.

**Table 2:** Resource use

Resource categories	Intervention	Control	
	Mean (SD)	Mean (SD)	Difference [95% CI]
Primary care (contacts)			
General practitioners	26.33 (16.66)	29.98 (20.11)	−3.65 [−7.61 to 0.31]
Medical specialists	2.74 (3.80)	2.51 (3.53)	0.23 [−0.56 to 1.02]
Therapists	3.22 (9.23)	3.01 (6.79)	0.21 [−1.52 to 1.95]
Other	4.29 (2.98)	4.46 (2.74)	−0.17 [−0.79 to 0.44]
Total primary care contacts	36.58 (22.61)	39.96 (23.27)	−3.38 [−8.30 to 1.55]
Secondary care			
Somatic hospital inpatient (admissions)	3.64 (3.59)	3.73 (3.24)	−0.09 [−0.82 to 0.64]
Somatic hospital outpatient (contacts)	34.45 (25.42)	35.14 (24.90)	−0.70 [−6.10 to 4.70]
Contact days	18.10 (19.40)	17.29 (15.37)	0.82 [−2.94 to 4.57]
Medicine (DDD)	46.42 (45.05)	52.11 (72.83)	−5.68 [−18.68 to 7.31]
Productivity loss			
Sick leave (weeks)	7.20 (19.21)	6.63 (15.44)	0.58 [−3.16 to 4.32]
Part time sick leave (weeks)	4.36 (14.82)	3.97 (12.30)	0.39 [−2.53 to 3.32]

*SD* standard deviation, *CI* confidence interval, *DDD* defined daily dose

### Costs

From a healthcare perspective, mean total costs, including intervention costs, were €40,208 (SD €28,780) in the intervention group and €41,190 (SD €28,380) in the control group, yielding a mean difference of −€980.56 (95% CI −€7120 to €5159) (Table 3). Expenditures related to primary care, secondary care, and prescription medicines were comparable between groups, with no substantial or statistically significant differences observed across individual categories.

**Table 3:** Costs (2024 €)

Resource categories	Intervention	Control	
	Mean (SD)	Mean (SD)	Difference [95% CI]
Primary care			
General practitioners	588.81 (413.21)	632.67 (488.40)	−43.87 [−140.53 to 52.80]
Medical specialists	213.84 (310.33)	208.46 (277.53)	5.38 [−57.43 to 68.19]
Therapists	89.60 (296.81)	68.87 (193.23)	20.73 [−33.48 to 74.94]
Other	175.89 (154.57)	200.68 (166.90)	−24.79 [−58.48 to 8.905]
Total primary care costs	1,068.14 (697.94)	1,110.69 (700.69)	−42.55 [−190.59 to 105.50]
Secondary care			
Somatic hospital inpatient (admissions)	26,219 (18,433)	26,231 (15,828)	−12.27 [−3701 to 3677]
Somatic hospital outpatient (contacts)	12,878 (14,901)	13,849 (16,835)	−970.86 [−4380 to 2438]
Total secondary care costs	39,097 (28,606)	40,080 (28,346)	−983.13 [−7095 to 5129]
Medicine costs	1,118 (1,670)	1,214 (1,621)	−96.56 [−442.47 to 249.35]
Total health care costs	40,165 (28,775)	41,190 (28,380)	−1026 [−7165 to 5113]
Intervention costs	45.13 (28.15)		
Total health care costs incl. intervention costs	40,208 (28,780)	41,190 (28,380)	−980.56 [−7120 to 5159]
Productivity loss (sick leave)	257.07 (640.66)	219.02 (463.94)	38.05 [−80.40 −156.50]
Total societal costs incl. intervention costs	40,467 (28,798)	41,409 (28,466)	−942.50 [−7093 to 5208]

*SD* standard deviation, *CI* confidence interval

From a societal perspective, total mean costs, including productivity loss, were €40,467 (SD €28,798) in the intervention group and €41,409 (SD €28,466) in the control group, corresponding to a difference of −942.50 (95% CI −€7093 to €5208). Across all domains, cost differences were small and associated with wide confidence intervals, indicating no significant differences between the intervention and control groups (Table 3).

### Cost-effectiveness

Health outcomes, measured in QALYs, were evaluated using both complete case and imputed case analyses. In the complete case analysis, the mean QALY over 3 years was 2.26 (SD 0.63) in the intervention group and 2.22 (SD 0.64) in the control group, corresponding to a mean difference of 0.044 (95% CI −0.099 to 0.187). The imputed case analysis yielded a mean QALY of 2.24 (SD 0.63) in the intervention group and 2.20 (SD 0.66) in the control group, with a mean difference of 0.028 (95% CI −0.106 to 0.163) (Table 4).

**Table 4:** Effectiveness

	Intervention			Total QALY (3 years)	Control			Total QALY (3 years)	Difference [95% CI]
	Baseline	Follow-up^1^	Follow-up^2^		Baseline	Follow-up^1^	Follow-up^2^		
	Mean (SD)	Mean (SD)	Mean (SD)	Mean (SD)	Mean (SD)	Mean (SD)	Mean (SD)	Mean (SD)	
Complete case analysis									
	*n: 160*	*n: 153*	*n: 155*		*n: 160*	*n: 148*	*n: 155*		
Utility (based on EQ-5D 5L*)	0.85 (0.16)	0.88 (0.22)	0.73 (0.39)		0.84 (0.18)	0.87 (0.24)	0.70 (0.42)		
		*n: 147*	*n: 145*	*n: 137*		*n: 146*	*n: 144*	*n: 137*	
QALY		0.73 (0.18)	1.48 (0.56)	2.26 (0.63)		0.73 (0.167)	1.44 (0.59)	2.22 (0.64)	0.044 [−0.099 to 0.187]
Imputed case analysis				*n: 168*				*n: 168*	
Utility (based on EQ-5D-5L*)	0.85 (0.15)	0.88 (0.22)	0.73 (0.37)		0.84 (0.18)	0.87 (0.23)	0.70 (0.40)		
QALY		0.74 (0.17)	1.51 (0.53)	2.24 (0.63)		0.73 (0.17)	1.47 (0.57)	2.20 (0.66)	0.028 [−0.106 to 0.163]

*QALY* quality adjusted life years, *SD* standard deviation, *CI* confidence interval

*EuroQol 5Dimensions 5 Levels Questionnaire

Based on the imputed case analysis, the incremental cost-effectiveness ratio (ICER) was −€35,048 per QALY gained from a healthcare perspective, and −€33,661 per QALY gained from a societal perspective. The negative ICER values indicate that the patient-led follow-up programme was dominant, resulting in lower costs and higher QALYs compared to standard follow-up. However, these differences were small and not statistically significant, with wide confidence intervals reflecting considerable uncertainty around the estimates.

**Fig. 1:**
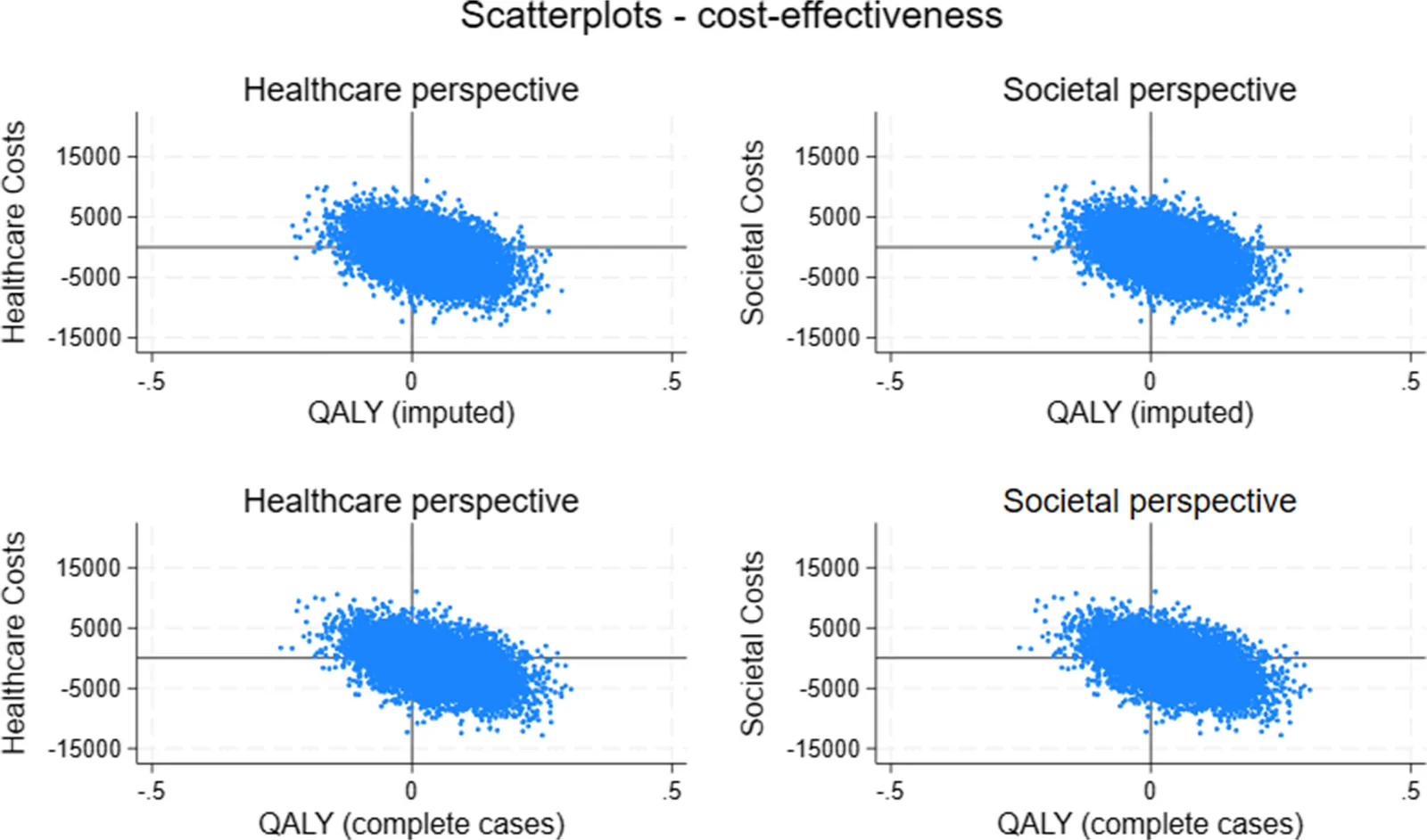
Scatterplots—cost-effectiveness

Scatterplots of 10,000 bootstrapped incremental cost-effectiveness pairs are shown in Fig. 1. The incremental pairs are distributed across all four quadrants of the cost-effectiveness plane, reflecting considerable statistical uncertainty regarding both incremental costs and effects. This pattern indicates that the patient-led intervention was associated with variable differences in costs and QALYs when compared to standard follow-up, with a substantial proportion of iterations indicating lower costs and improved outcomes, but also many suggesting the opposite or neutral effects.

**Fig. 2:**
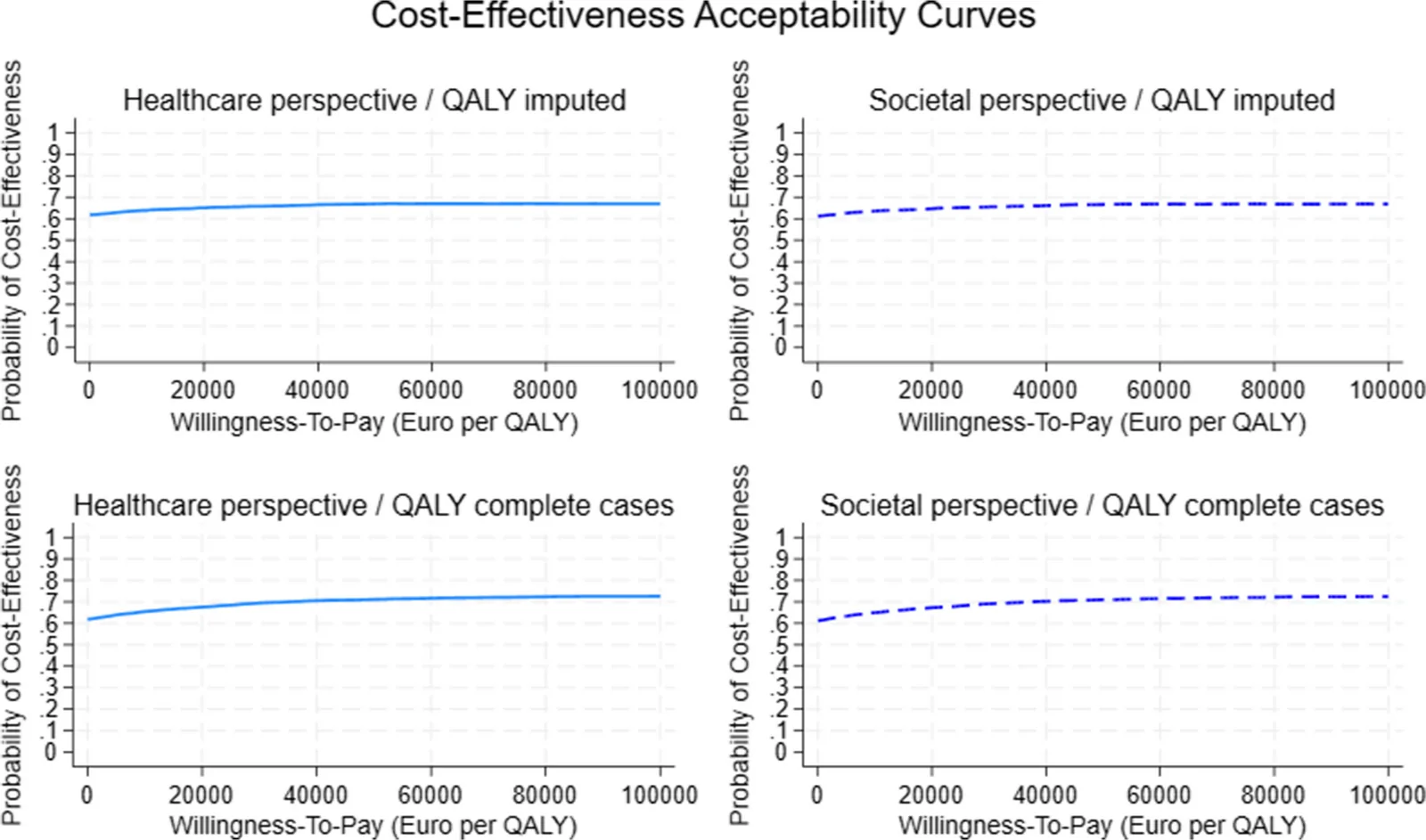
Cost-effectiveness acceptability curves

The cost-effectiveness acceptability curves (Fig. 2) further illustrate this uncertainty. Across both healthcare and societal perspectives, and for both imputed and complete case analyses, the probability that the patient-led programme is cost-effective remained below 70% at a willingness-to-pay threshold of €30,000 per QALY gained. These findings underscore the presence of wide uncertainty and indicate that, at conventional willingness-to-pay thresholds, the likelihood of the intervention being cost-effective is modest.

### Sensitivity analyses

Subgroup analyses were conducted based on surgical approach and pre-treatment status using imputed cases (Supplementary file 2).

Subgroup analyses by surgical approach and pre-treatment status indicated variation in the potential for cost-effectiveness. For patients undergoing total mesorectal excision, the patient-led programme was on average more costly and less effective than standard follow-up, although estimates were highly uncertain. In contrast, for partial mesorectal excision and abdomino-perineal resection, the intervention was associated with lower costs and slightly higher QALYs, suggesting dominance in these subgroups.

When stratified by pre-treatment status, no meaningful incremental QALY was observed among patients without neoadjuvant treatment, while for those receiving neoadjuvant treatment the intervention was associated with higher QALYs at modest additional costs, resulting in an incremental cost per QALY well below conventional willingness-to-pay thresholds.

The cost-effectiveness acceptability curves (Supplementary file 3) illustrate that the probability of cost-effectiveness was consistently higher for partial mesorectal excision and abdomino-perineal resection compared to total mesorectal excision and increased with willingness-to-pay thresholds among patients who had received neoadjuvant treatment. At €30,000 per QALY, the probability of cost-effectiveness was approximately 70–75% for partial mesorectal excision and abdomino-perineal resection, around 70% for patients with neoadjuvant treatment, and below 40% for total mesorectal excision.

## Discussion

### Principal findings

This multicentre, randomised trial represents the first long-term economic evaluation of patient-led follow-up after curative-intent rectal cancer surgery. Over a 3-year time horizon, the patient-led model resulted in comparable health outcomes to standard surgeon-led follow-up, with mean QALYs showing no statistically significant difference between groups. Mean total costs were lower for the patient-led programme, although these differences were not statistically significant.

In a previous analysis of 1-year follow-up data from the FURCA trial, audit data from electronic medical records were used to identify all hospital contacts registered within the treating department in both trial arms during the first postoperative year. The total number of hospital contacts was similar between groups; however, patients in the intervention arm had significantly fewer physician-led contacts and a higher number of nurse-led contacts, reflecting the intended design of the intervention. The composition of contacts differed significantly between groups, indicating a more needs-based pattern of follow-up in the intervention arm. This 1-year analysis also assessed protocol adherence and documented reasons for deviations from the assigned follow-up model [37]. In the main analysis, the incremental cost-effectiveness ratio (ICER) was −€35,048 per QALY gained from a healthcare perspective, indicating that the patient-led follow-up programme was associated with both lower costs and higher QALYs compared to standard follow-up (i.e., the intervention was dominant). However, the differences were small, and the associated confidence intervals were wide, reflecting considerable statistical uncertainty. The probability of the intervention being cost-effective at a willingness-to-pay threshold of €30,000 per QALY remained below 70%.

Sensitivity analyses suggested some variation in cost-effectiveness by surgical approach and pre-treatment status. The intervention tended to be more favourable among patients undergoing PME and APR, and among those who received neoadjuvant treatment, while being less favourable for patients undergoing TME or those who did not receive neoadjuvant treatment. This may reflect the clinical variation between the sub-groups in patient follow-up needs. However, all subgroup estimates were characterised by wide confidence intervals and should be interpreted with caution.

### Comparison with previous literature

Our findings extend a limited, yet evolving, body of evidence on the cost-effectiveness of alternative follow-up strategies in colorectal cancer survivorship. Previous studies have primarily evaluated nurse-led or GP-led models, often within non-randomised designs or with limited follow-up [14, 38, 39]. For instance, Vos et al. demonstrated that GP-led care after colon cancer may be cost-effective with respect to disease-specific, but not generic, quality of life [16], whereas Qaderi et al. identified short-term cost savings with remote follow-up, though their study did not include a full health economic evaluation or long-term outcomes [17]. In endometrial cancer, patient-initiated follow-up has shown favourable cost and time savings; however, the transferability of these findings to rectal cancer is uncertain due to differences in late-effect profiles and healthcare needs [20].

Systematic reviews have underscored both the potential and the limitations of the current evidence base. Meregaglia and Cairns concluded that less intensive, primary care-based, and risk-adapted follow-up schemes are increasingly supported in colorectal cancer, though methodological heterogeneity and limited long-term data remain important barriers to definitive guidance [15]. Similarly, Barbieri et al. found that intensive follow-up for colorectal cancer may be cost-effective in selected contexts, but heterogeneity in study designs and outcome measures makes generalisation difficult [14].

Cost-effectiveness modelling studies, such as those by Castelli et al. and Takayama et al., further indicate that mean cost and incremental net benefit may differ by follow-up strategy, cancer stage, and surveillance interval, but often fail to show that one strategy is definitively superior across all patient groups [40, 41]. Other evaluations emphasise that surveillance protocols should be tailored to recurrence risk and that risk-adapted approaches may be most efficient [42, 43]. Studies of remote surveillance, such as Siddika et al., confirm the safety, high patient satisfaction, and substantial cost savings of these models, but typically focus on short-term outcomes and may not fully capture longer-term health system or patient-centred effects [44].

Research on nurse-led follow-up, as reviewed by Wille-Jørgensen and Balleby, has shown nurse-led care to be non-inferior to physician-led follow-up in recurrence detection and patient satisfaction, although resource use and cost-effectiveness comparisons require further research [39]. Furthermore, Staib et al. and Di Cristofaro et al. have advocated for surveillance protocols that incorporate risk stratification and cost-effectiveness principles, reinforcing the trend towards more individualised and efficient survivorship care [38, 43].

Despite these advances, high-quality, randomised evidence on patient-initiated follow-up in rectal cancer remains scarce. The present study provides the first randomised, individual-level, long-term data on patient-initiated follow-up after rectal cancer. Importantly, the FURCA trial employed a societal perspective, incorporating both direct healthcare expenditures and productivity losses, in contrast to most previous evaluations that focused on health system costs alone. This comprehensive approach is particularly salient for survivorship populations, where long-term morbidity may influence both service utilisation and labour market participation.

### Interpretation of health economic findings

The absence of significant cost or QALY differences between follow-up models suggests that the relocation of routine surveillance from surgeons to specialist nurses, combined with patient self-referral and structured education, did not materially alter either resource use or patient outcomes over 3 years. While the intervention led to a slight reduction in primary care contacts and a minor cost advantage, these effects were modest and surrounded by considerable statistical uncertainty.

From a health economic perspective, the results indicate that patient-led follow-up is at least cost-neutral, without evidence of harm or excess resource use. However, the probability of cost-effectiveness under prevailing willingness-to-pay thresholds indicates that, within the constraints of this study and context, patient-led models are unlikely to generate substantial economic gains when QALYs and direct costs are the primary metrics. It is plausible that unmeasured dimensions, such as patient empowerment, informal caregiving, or reduced burden on specialist services, may offer additional value not fully captured by the cost-utility framework.

### Strengths and limitations

A major strength of this evaluation is the use of population-based, high-quality registry data, minimising the risk of recall or reporting bias and ensuring comprehensive capture of health service utilisation and productivity losses. The randomised, multicentre design enhances internal validity and reflects routine practice in Danish cancer follow-up. Cancer treatment and follow-up in Denmark is standardised nationally and follows national guidelines, reducing the risk of local variation in follow-up practice [23] We believe that the study population is representative of the general RC patient population, as the sample included both rural and urban municipalities.

Economic analyses were prespecified, and statistical uncertainty was rigorously addressed through non-parametric bootstrapping and probabilistic sensitivity analysis, following international best practices.

Nevertheless, limitations must be acknowledged. First, although relevant to a comprehensive societal perspective, municipal services and informal care were not included in the main analysis due to lack of systematic data across all participating municipalities. These services, such as home nursing, rehabilitation, and informal caregiving, may have been affected by the intervention but were not uniformly recorded in national registers and could not be reliably incorporated in the evaluation. This may underestimate total societal costs, particularly for patients with greater late-effect burdens, and may mask potential cost-shifting between healthcare sectors. Second, the EQ-5D-5L instrument, while widely accepted for economic evaluation, may not fully capture all relevant dimensions of cancer survivorship, such as psychosocial adaptation or cancer-specific symptoms. Third, the trial was powered for clinical rather than economic endpoints, which limits the precision of subgroup estimates and increases the risk of type II error in the cost-utility outcomes. This limitation is further compounded by the skewed distribution of cost and utility data, which reduces statistical efficiency and makes detection of statistically significant differences more challenging despite appropriate analytical methods. This was particularly evident in the sensitivity analyses, where small subgroup sizes were reflected in wide confidence intervals. In addition, the economic evaluation was conducted over the trial follow-up period rather than a lifetime horizon. While it is appropriate for a follow-up intervention not expected to influence long-term survival, this limits insight into the persistence of cost and quality-of-life effects beyond the observed period, which could be explored in future long-term observational studies. Finally, the Danish setting, with universal coverage and robust care coordination, facilitates timely access to specialist care and reduces patient barriers to self-referral. Conversely, in healthcare systems with different organisational or financing models, patient-initiated follow-up may be inhibited by direct or indirect costs, insurance restrictions, or fragmented care structures. This must be taken into account when considering wider policy implications and implementation in other healthcare systems.

### Implications for policy and practice

The FURCA trial offers important insights for health policy and the organisation of survivorship care. The finding that patient-led follow-up is broadly equivalent in terms of costs and QALYs to conventional models, with no evidence of increased resource use, suggests that such approaches may be safely adopted where there is policy interest in patient empowerment and system flexibility. Analyses from the same trial have reported significantly higher levels of patient satisfaction, perceived involvement in care, without any measurable adverse clinical outcomes from the intervention [37].

Notably, the absence of cost savings or significant QALY gains implies that decisions to implement patient-led models should also consider non-economic factors, including patient preference, access, and feasibility within local organisational contexts. While a more responsive approach such as patient-led follow-up could require additional resource use in some cases, the goal is not solely cost reduction but optimal allocation—delivering the right intervention, at the right time, to the right patient [37]. In this sense, a cost-neutral but more responsive system could represent an efficiency gain in terms of both health outcomes and equity.

These findings should also be considered in the broader context of current healthcare transformation agendas emphasising a shift of care delivery away from specialist hospital settings and into primary care or community-based services. Such a reorganisation is aimed at optimising specialist capacity, improving accessibility, and supporting continuity of care closer to patients’ everyday environments. Patient-led follow-up models, particularly when coordinated by skilled nursing staff, may provide a central element within this strategic direction, by offering tailored and specialised care without compromising safety or increasing costs.

Since the initiation of the FURCA trial, follow-up care has shifted from a narrow focus on recurrence detection to broader management of late adverse effects, as prioritised in the Danish Cancer Plan V [45]. In the FURCA study regions, structured screening and targeted management of late adverse effects have now been implemented. While this may influence patterns of resource utilisation and staff time, the organisational framework remains predominantly patient-led and relies heavily on specialist nursing staff. The cost implications of such a shift are yet to be fully evaluated, but the continuity in approach suggests that lessons from this trial remain directly relevant.

### Implications for future research

Further studies should extend follow-up beyond 3 years to assess long-term survivorship outcomes, including late-onset morbidity and health system costs. Efforts to systematically capture municipal, social, and informal care are warranted to more fully reflect the societal impact of survivorship care models. In addition, patient-reported outcomes beyond generic QoL, including satisfaction, empowerment, and self-efficacy, should be prioritised. Economic evaluations in other health system contexts will also be essential to assess transferability and inform international guideline development.

## Conclusions

Patient-led follow-up after rectal cancer surgery was not associated with significant gains in QALYs or cost savings relative to standard surgeon-led follow-up and was unlikely to be cost-effective at conventional willingness-to-pay thresholds. While cost-neutrality and comparable outcomes support the safety of patient-led models, further research should examine broader patient-centred outcomes, longer-term effects, and the full spectrum of resource implications to inform future survivorship care policy and practice.

## Supplementary information

Below is the link to the electronic supplementary material.

ESM 1 (PDF 101 KB)

ESM 2 (DOCX 24.4 KB)

ESM 3 (DOCX 74.8 KB)

## Data Availability

Due to the GDPR regulations data are not available, yet questions regarding aggregated data may be addressed to the corresponding author.

## References

[1] Wong MCS, Huang J, Lok V, Wang J, Fung F, Ding H, et al. Differences in incidence and mortality trends of colorectal cancer worldwide based on sex, age, and anatomic location. Clin Gastroenterol Hepatol. 2021;19(5):955–66.e61. 10.1016/j.cgh.2020.02.026. 32088300

[2] Sung H, Ferlay J, Siegel RL, Laversanne M, Soerjomataram I, Jemal A, et al. Global cancer statistics 2020: GLOBOCAN estimates of incidence and mortality worldwide for 36 cancers in 185 countries. CA Cancer J Clin. 2021;71(3):209–49. 10.3322/caac.21660. 33538338

[3] Regionernes Kliniske Kvalitetsprogram (RKKP). Dansk Kolorektal Cancer Database Årsrapport 1. januar 2023 - 31. december 2023 [Danish Colorectal Cancer Database Annual Report January 1 to December 31 2023][Internet]. Aarhus: Regionernes Kliniske Kvalitets program; 2024 [cited 2026 January 28]. Available from: https://data-sundk.dk/aarsrapporter/dccg/

[4] Bülow S, Harling H, Iversen LH, Ladelund S. Improved survival after rectal cancer in Denmark. Colorectal Dis. 2010;12(7):e37-42. 10.1111/j.1463-1318.2009.02012.x. 19614669

[5] Cheng L, Eng C, Nieman LZ, Kapadia AS, Du XL. Trends in colorectal cancer incidence by anatomic site and disease stage in the United States from 1976 to 2005. Am J Clin Oncol. 2011;34(6):573–80. 10.1097/COC.0b013e3181fe41ed. 21217399

[6] Bastiaenen VP, Hovdenak Jakobsen I, Labianca R, Martling A, Morton DG, Primrose JN, et al. Consensus and controversies regarding follow-up after treatment with curative intent of nonmetastatic colorectal cancer: a synopsis of guidelines used in countries represented in the European Society of Coloproctology. Colorectal Dis. 2019;21(4):392–416. 10.1111/codi.14503. 30506553

[7] Juul T, Bräuner AB, Drewes AM, Emmertsen KJ, Krogh K, Laurberg S, et al. Systematic screening for late sequelae after colorectal cancer - a feasibility study. Colorectal Dis. 2021. 10.1111/codi.15519. 33420746

[8] Mikkelsen AH, Thyø A, Seyer-Hansen AD, Drewes AM, Laursen BS, Christensen P, et al. Sexual dysfunction after colorectal and anal cancer-treatment in nurse-led sexological clinics. Colorectal Dis. 2025;27(2):e70018. 10.1111/codi.70018. PMC11790510 39900565

[9] Mekhael M, Kristensen H, Borre M, Drewes AM, Emmertsen KJ, Fassov J, et al. Treatment of low anterior resection syndrome in specialized multidisciplinary late sequelae clinics: a prospective cohort study. Ann Surg. 2025. 10.1097/sla.0000000000006714. 40168548

[10] Mekhael M, Larsen HM, Lauritzen MB, Thorlacius-Ussing O, Laurberg S, Krogh K, et al. Bowel dysfunction following pelvic organ cancer: a prospective study on the treatment effect in nurse-led late sequelae clinics. Acta Oncol. 2023;62(1):70–9. 10.1080/0284186x.2023.2168214. 36757368

[11] Batehup L, Porter K, Gage H, Williams P, Simmonds P, Lowson E, et al. Follow-up after curative treatment for colorectal cancer: longitudinal evaluation of patient initiated follow-up in the first 12 months. Support Care Cancer. 2017;25(7):2063–73. 10.1007/s00520-017-3595-x. PMC5445145 28197848

[12] Jefford M, Emery J, Grunfeld E, Martin A, Rodger P, Murray AM, et al. SCORE: Shared care of Colorectal cancer survivors: protocol for a randomised controlled trial. Trials. 2017;18(1):506. 10.1186/s13063-017-2245-4. PMC5663101 29084595

[13] Jefford M, Gough K, Drosdowsky A, Russell L, Aranda S, Butow P, et al. A randomized controlled trial of a nurse-led supportive care package (SurvivorCare) for survivors of colorectal cancer. Oncologist. 2016;21(8):1014–23. 10.1634/theoncologist.2015-0533. PMC4978555 27306909

[14] Barbieri M, Richardson G, Paisley S. The cost-effectiveness of follow-up strategies after cancer treatment: a systematic literature review. Br Med Bull. 2018;126(1):85–100. 10.1093/bmb/ldy011. 29659715

[15] Meregaglia M, Cairns J. Economic evaluations of follow-up strategies for cancer survivors: a systematic review and quality appraisal of the literature. Expert Rev Pharmacoecon Outcomes Res. 2015;15(6):913–29. 10.1586/14737167.2015.1087316. 26449255

[16] Vos JAM, El Alili M, Duineveld LAM, Wieldraaijer T, Wind J, Sert E, et al. Cost-effectiveness of general practitioner- versus surgeon-led colon cancer survivorship care: an economic evaluation alongside a randomised controlled trial. J Cancer Surviv. 2023. 10.1007/s11764-023-01383-4. PMC11324670 37097550

[17] Qaderi SM, Swartjes H, Vromen H, Bremers AJA, Custers JAE, de Wilt JHW. Acceptability, quality of life and cost overview of a remote follow-up plan for patients with colorectal cancer. Eur J Surg Oncol. 2021. 10.1016/j.ejso.2020.12.018. 33423826

[18] Bhimani N, Wong GY, Molloy C, Dieng M, Hugh TJ. Cost of colorectal cancer by treatment type from different health economic perspectives: a systematic review. Eur J Surg Oncol. 2022. 10.1016/j.ejso.2022.06.023. 35787341

[19] Vahdatimanesh Z, Zendehdel K, Kbari Sari AA, Farhan F, Nahvijou A, Delavari A, et al. Economic burden of colorectal cancer in Iran in 2012. Med J Islam Repub Iran. 2017;31:115. 10.14196/mjiri.31.115. PMC6014770 29951416

[20] Luqman I, Wickham-Joseph R, Cooper N, Boulter L, Patel N, Kumarakulasingam P, et al. Patient-initiated follow-up for low-risk endometrial cancer: a cost-analysis evaluation. Int J Gynecol Cancer. 2020;30(7):1000–4. 10.1136/ijgc-2019-001074 32522772

[21] Hovdenak Jakobsen I, Juul T, Bernstein I, Christensen P, Jensen FS, Johansen C, et al. Follow-up after rectal cancer: developing and testing a novel patient-led follow-up program. Study protocol. Acta Oncol. 2017;56(2):307–13. 10.1080/0284186x.2016.1267400. 28068158

[22] Husereau D, Drummond M, Petrou S, Carswell C, Moher D, Greenberg D, et al. Consolidated Health Economic Evaluation Reporting Standards (CHEERS) statement. Eur J Health Econ. 2013;14(3):367–72. 10.1007/s10198-013-0471-6 23526140

[23] Sundhedsstyrelsen. Opfølgningsprogram for tyk-og endetarmskræft [Follow-up Program after Colorectal Cancer]. Copenhagen: Sundhedsstyrelsen; 2015 [cited 2025 January 28]. Available from: https://sundhedsstyrelsen.dk/da/udgivelser/2015/%7E/media/95F5F42112AF4463A509D6858DDDAD52.ashx?m=.pdf

[24] Danish Colorectal Cancer Group (DCCG). DCCG’s nationale retningslinjer for diagnostik og behandling af kolorektal cancer: Postoperativ Kontrol [National Guidelines for diagnostics and treatment of colorectal cancer: Postoperative Control]. Copenhagen; 2012 [cited 2025 January 28]. Available from: https://dccg.dk/wp-content/uploads/2023/06/Postoperativ-kontrol.pdf

[25] Jensen CE, Sørensen SS, Gudex C, Jensen MB, Pedersen KM, Ehlers LH. The Danish EQ-5D-5L value set: a hybrid model using cTTO and DCE data. Appl Health Econ Health Policy. 2021;19(4):579–91. 10.1007/s40258-021-00639-3 PMC8270796 33527304

[26] Andersen JS, Olivarius NF, Krasnik A. The Danish National Health Service Register. Scand J Public Health. 2011;39(7 Suppl):34–7. 10.1177/1403494810394718 21775348

[27] Lynge E, Sandegaard JL, Rebolj M. The Danish National Patient Register. Scand J Public Health. 2011;39(7 Suppl):30–3. 10.1177/1403494811401482 21775347

[28] Kildemoes HW, Sørensen HT, Hallas J. The Danish National Prescription Registry. Scand J Public Health. 2011;39(7 Suppl):38–41. 10.1177/1403494810394717 21775349

[29] Stapelfeldt CM, Jensen C, Andersen NT, Fleten N, Nielsen CV. Validation of sick leave measures: self-reported sick leave and sickness benefit data from a Danish national register compared to multiple workplace-registered sick leave spells in a Danish municipality. BMC Public Health. 2012;12:661. 10.1186/1471-2458-12-661 PMC3511193 22894644

[30] Drummond MFSM. Claxton K, Stoddart GL. Torrance GW Methods for the economic evaluation of health care programmes.: Oxford University Press; 2015.

[31] Faria R, Gomes M, Epstein D, White IR. A guide to handling missing data in cost-effectiveness analysis conducted within randomised controlled trials. Pharmacoeconomics. 2014;32(12):1157–70. 10.1007/s40273-014-0193-3 PMC4244574 25069632

[32] Manca A, Hawkins N, Sculpher MJ. Estimating mean QALYs in trial-based cost-effectiveness analysis: the importance of controlling for baseline utility. Health Econ. 2005;14(5):487–96. 10.1002/hec.944. 15497198

[33] Desgagné A, Castilloux AM, Angers JF, LeLorier J. The use of the bootstrap statistical method for the pharmacoeconomic cost analysis of skewed data. Pharmacoeconomics. 1998;13(5 Pt 1):487–97. 10.2165/00019053-199813050-00002. 10180748

[34] Briggs A, Nixon R, Dixon S, Thompson S. Parametric modelling of cost data: some simulation evidence. Health Econ. 2005;14(4):421–8. 10.1002/hec.941. 15685641

[35] Briggs AH, Wonderling DE, Mooney CZ. Pulling cost-effectiveness analysis up by its bootstraps: a non-parametric approach to confidence interval estimation. Health Econ. 1997;6(4):327–40. 10.1002/(sici)1099-1050(199707)6:4<327::aid-hec282>3.0.co;2-w. 9285227

[36] Hovdenak I, Thaysen HV, Bernstein IT, Christensen P, Hauberg A, Iversen LH, et al. Quality of life and symptom burden after rectal cancer surgery: a randomised controlled trial comparing patient-led versus standard follow-up. J Cancer Surviv. 2023. 10.1007/s11764-023-01410-4. PMC11424718 37395934

[37] Hovdenak Jakobsen I, Vind Thaysen H, Laurberg S, Johansen C, Juul T. Patient-led follow-up reduces outpatient doctor visits and improves patient satisfaction. One-year analysis of secondary outcomes in the randomised trial Follow-Up after Rectal CAncer (FURCA). Acta Oncol. 2021;60(9):1130–9. 10.1080/0284186x.2021.1950924. 34238100

[38] Staib L, Link KH, Beger HG. Follow-up in colorectal cancer: cost-effectiveness analysis of established and novel concepts. Langenbecks Arch Surg. 2000;385(6):412–20. 10.1007/s004230000144. 11127527

[39] Wille-Jørgensen P, Balleby L. Follow-up in colorectal cancer: questions to be answered. Colorectal Dis. 2011;13(9):959–60. 10.1111/j.1463-1318.2011.02716.x. 21848730

[40] Castelli C, Combescure C, Foucher Y, Daures JP. Cost-effectiveness analysis in colorectal cancer using a semi-Markov model. Stat Med. 2007;26(30):5557–71. 10.1002/sim.3112. 18058847

[41] Takayama Y, Tsukamoto S, Kudose Y, Takamizawa Y, Moritani K, Esaki M, et al. Cost-effectiveness of surveillance intervals after curative resection of colorectal cancer. Jpn J Clin Oncol. 2024;54(6):637–46. 10.1093/jjco/hyae018. 38376792

[42] Alsowaina KN, Schlachta CM, Alkhamesi NA. Cost-effectiveness of current approaches in rectal surgery. Ann Med Surg (Lond). 2019;45:36–9. 10.1016/j.amsu.2019.07.004. PMC6639648 31360458

[43] Di Cristofaro L, Scarpa M, Angriman I, Perissinotto E, Ruffolo C, Frego M, et al. Cost-effectiveness analysis of postoperative surveillance protocols following radical surgery for colorectal cancer. Acta Chir Belg. 2012;112(1):24–32. 10.1080/00015458.2012.11680791. 22442906

[44] Siddika A, Tolia-Shah D, Pearson TE, Richardson NG, Ross AH. Remote surveillance after colorectal cancer surgery: an effective alternative to standard clinic-based follow-up. Colorectal Dis. 2015;17(10):870–5. 10.1111/codi.12970. 25851058

[45] Indenrigs- og Sundhedsministeriet. Et bedre liv med og efter kræft - Kræftplan V [Improved quality of life for cancer patients and camcer survivors - Cancer Care Plan V]. Copenhagen; 2025 [cited 2026 January 28]. Available from: https://www.ism.dk/Media/638835090309035273/Et-bedre-liv-med-og-efter-kraeft-kraeftplan-V-TILG.pdf

