# Supplementary material for: Cost-Utility analysis of Patient-Led Follow-Up after Rectal Cancer Compared to Standard Follow-Up: A three-year follow-up of the FURCA Randomised Controlled Trial
Source: J Cancer Surviv. 2026 Jan 29;20(Suppl 2):1467–78. doi: 10.1007/s11764-026-01976-9 (PMC13744497; doi:10.1007/s11764-026-01976-9)
Supplement: Supplementary file 1 [file 11764_2026_1976_MOESM1_ESM.pdf]

CHEERS 2022 Checklist

**Title**			
Title	1	Identify the study as an economic evaluation and specify the interventions being compared.	Title, 1st page
**Abstract**			
Abstract	2	Provide a structured summary that highlights context, key methods, results, and alternative analyses.	Abstract: 2nd page
**Introduction**			
Background and objectives	3	Give the context for the study, the study question, and its practical relevance for decision making in policy or practice.	Introduction section
Methods			
**Health economic analysis plan**	**4**	**Indicate whether a health economic analysis plan was developed and where available.**	**n/a**
Study population	5	Describe characteristics of the study population (such as age range, demographics, socioeconomic, or clinical characteristics).	Methods section, sub-sections: participants and allocation; intervention
Setting and location	6	Provide relevant contextual information that may influence findings.	Methods section, sub-section: Study design and setting
Comparators	7	Describe the interventions or strategies being compared and why chosen.	Methods section, sub-section: intervention
Perspective	8	State the perspective(s) adopted by the study and why chosen.	Methods section, sub-section: Time horizon and perspective
Time horizon	9	State the time horizon for the study and why appropriate.	Methods section, sub-section: Time horizon and perspective

(continued)

Discount rate	10	Report the discount rate(s) and reason chosen.	Methods section, sub-section: Measurement of outcomes
Selection of outcomes	11	Describe what outcomes were used as the measure(s) of benefit(s) and harm(s).	Methods section, sub-section: Measurement of outcomes
Measurement of outcomes	12	Describe how outcomes used to capture benefit(s) and harm(s) were measured.	Methods section, sub-sections: Measurement of outcomes; Resource use and costing
Valuation of outcomes	13	Describe the population and methods used to measure and value outcomes.	Methods section, sub-sections: Measurement of outcomes
Measurement and valuation of resources and costs	14	Describe how costs were valued.	Methods section, sub-sections: Resource use and costing
Currency, price date, and conversion	15	Report the dates of the estimated resource quantities and unit costs, plus the currency and year of conversion.	Methods section, sub-sections: Resource use and costing (last paragraph).
Rationale and description of model	16	If modelling is used, describe in detail and why used. Report if the model is publicly available and where it can be accessed.	N/A
Analytics and assumptions	17	Describe any methods for analysing or statistically transforming data, any extrapolation methods, and approaches for validating any model used.	Methods section, sub-section: Statistical and economic analysis
Characterising heterogeneity	18	Describe any methods used for estimating how the results of the study vary for subgroups.	Methods section, sub-section: Subgroup and sensitivity analyses

(continued)

Characterising distributional effects	19	Describe how impacts are distributed across different individuals or adjustments made to reflect priority populations.	N/A
Characterising uncertainty	20	Describe methods to characterise any sources of uncertainty in the analysis.	Methods section, sub-section: Subgroup and sensitivity analyses
Approach to engagement with patients and others affected by the study	21	Describe any approaches to engage patients or service recipients, the general public, communities, or stakeholders (such as clinicians or payers) in the design of the study.	N/A
**Results**			
Study parameters	22	Report all analytic inputs (such as values, ranges, references) including uncertainty or distributional assumptions.	Tables 1, 2, 3 ; Results section
Summary of main results	23	Report the mean values for the main categories of costs and outcomes of interest and summarise them in the most appropriate overall measure.	Results, sub-sections: Costs, cost-effectiveness, Table 4
Effect of uncertainty	24	Describe how uncertainty about analytic judgments, inputs, or projections affect findings. Report the effect of choice of discount rate and time horizon, if applicable.	Results, sub-section: Sensitivity anlyses
Effect of engagement with patients and others affected by the study	25	Report on any difference patient/service recipient, general public, community, or stakeholder involvement made to the approach or findings of the study	Table 5; Fig 1 and 2; Results - sub-section 'sensitivity analyses'
Discussion			

(continued)

Study findings, limitations, generalisability, and current knowledge	26	Report key findings, limitations, ethical or equity considerations not captured, and how these could affect patients, policy, or practice.	Discussion, sub-sections: principal findings; Strength and limitations; Implications for policy and practice
Other relevant information			
Source of funding	27	Describe how the study was funded and any role of the funder in the identification, design, conduct, and reporting of the analysis	Acknowledgements, last page
Conflicts of interest	28	Report authors conflicts of interest according to journal or International Committee of Medical Journal Editors requirements.	Title page

From: Husereau D, Drummond M, Augustovski F, et al. Consolidated Health Economic Evaluation Reporting Standards 2022 (CHEERS 2022) Explanation and Elaboration: A Report of the ISPOR CHEERS II Good Practices Task Force. Value Health 2022;25. doi:10.1016/j.jval.2021.10.008
