# Supplementary material for: Cost-Utility analysis of Patient-Led Follow-Up after Rectal Cancer Compared to Standard Follow-Up: A three-year follow-up of the FURCA Randomised Controlled Trial
Source: J Cancer Surviv. 2026 Jan 29;20(Suppl 2):1467–78. doi: 10.1007/s11764-026-01976-9 (PMC13744497; doi:10.1007/s11764-026-01976-9)
Supplement: Supplementary file 2 [file 11764_2026_1976_MOESM2_ESM.docx]

| Supplementary file 2.  Sensitivity analyses |  |  |  |
| --- | --- | --- | --- |
|  | Intervention | Control |  |
|  | Mean (SD) | Mean (SD) | Difference [95% CI] |
| **Surgery Type** |  |  |  |
| Total Mesorectal Excision | *n: 66* | *n:76* |  |
| QALY^*^ | 2.29 (0.66) | 2.32 (0.53) | -0.07 [-0.27 ; 0.12] |
| Total health care costs | 44,558 (29,576) | 43,338 (30,357) | 1,220 [-8,546 ; 10,987] |
| Total societal costs | 44,880 (29,666) | 43,642 (30,497) | 1,238 [-8,564 ; 11,040] |
| Partial Mesorectal Excision | *n:40* | *n:31* |  |
| QALY^*^ | 2.35 (0.61) | 2.24 (0.66) | 0.11 [-0.18 ; 0.40] |
| Total health care costs | 33,513 (24,353) | 36,917 (20,368) | -3,404 [-13,584 ; 6,776] |
| Total societal costs | 33,708 (24,380) | 37,139 (20,442) | -3,431 [-13,639 ; 6,776] |
| Abdomino-perineal Resection | *n: 62* | *n: 61* |  |
| QALY^*^ | 2.02 (0.77) | 2.17 (0.61) | 0.14 [-0.10 ; 0.39] |
| Total health care costs | 39,902 (30,105) | 40,687 (29,443) | -785 [-11,303 ; 9,733] |
| Total societal costs | 40,130 (30,026) | 40,798 (29,458) | -668 [-11,174 ; 9,837] |
| **Pre-treatment** |  |  |  |
| No pre-treatment recieved | *n: 128* | *n: 129* |  |
| QALY^*^ | 2.29 (0.62) | 2.29 (0.59) | -0.00 [-0.15 ; 0.15] |
| Total health care costs | 38,990 (26,449) | 41,372 (29,658) | -2,381 [-9,180 ; 4,417] |
| Total societal costs | 39,163 (26,506) | 41,616 (29,759) | -2,453 [-9,273 ; 4,367] |
| Pre-treatment recieved | *n: 40* | *n: 39* |  |
| QALY^*^ | 2.08 (0.66) | 1.89 (0.78) | 0.20 [-0.12 ; 0.52] |
| Total health care costs | 44,112 (35,330) | 40,590 (24,005) | 3,523 [-9,626 ; 16,671] |
| Total societal costs | 44,638 (35,213) | 40,725 (24,033) | 3,913 [-9,210 ; 17,036] |
| *QALY: Qality Adjusted Life Years, SD:Standard Deviation, CI: Confidence Interval* | | | |
| ** QALY results are based on imputed cases* |  |  |  |
