# Supplementary material for: Cost-Utility analysis of Patient-Led Follow-Up after Rectal Cancer Compared to Standard Follow-Up: A three-year follow-up of the FURCA Randomised Controlled Trial
Source: J Cancer Surviv. 2026 Jan 29;20(Suppl 2):1467–78. doi: 10.1007/s11764-026-01976-9 (PMC13744497; doi:10.1007/s11764-026-01976-9)
Supplement: Supplementary file 3 [file 11764_2026_1976_MOESM3_ESM.docx]

Supplementary file 3:

Cost-Effectiveness Acceptability Curves.

Healthcare sector perspective / QALY imputed
